# Space-Dependent Glia–Neuron Interplay in the Hippocampus of Transgenic Models of β-Amyloid Deposition

**DOI:** 10.3390/ijms21249441

**Published:** 2020-12-11

**Authors:** Daniele Lana, Filippo Ugolini, Maria Grazia Giovannini

**Affiliations:** 1 Department of Health Sciences, Section of Clinical Pharmacology and Oncology, University of Florence, Viale Pieraccini 6, 50139 Firenze, Italy; daniele.lana@unifi.it; 2 Department of Health Sciences, Section of Anatomopathology, University of Florence, Viale Pieraccini 6, 50139 Firenze, Italy; filippo.ugolini@unifi.it

**Keywords:** TgCRND8, amyloid plaques, CA1 hippocampus, CA3 hippocampus, triads, confocal microscopy, astrocytes, microglia, clasmatodendrosis

## Abstract

This review is focused on the description and discussion of the alterations of astrocytes and microglia interplay in models of Alzheimer’s disease (AD). AD is an age-related neurodegenerative pathology with a slowly progressive and irreversible decline of cognitive functions. One of AD’s histopathological hallmarks is the deposition of amyloid beta (Aβ) plaques in the brain. Long regarded as a non-specific, mere consequence of AD pathology, activation of microglia and astrocytes is now considered a key factor in both initiation and progression of the disease, and suppression of astrogliosis exacerbates neuropathology. Reactive astrocytes and microglia overexpress many cytokines, chemokines, and signaling molecules that activate or damage neighboring cells and their mutual interplay can result in virtuous/vicious cycles which differ in different brain regions. Heterogeneity of glia, either between or within a particular brain region, is likely to be relevant in healthy conditions and disease processes. Differential crosstalk between astrocytes and microglia in CA1 and CA3 areas of the hippocampus can be responsible for the differential sensitivity of the two areas to insults. Understanding the spatial differences and roles of glia will allow us to assess how these interactions can influence the state and progression of the disease, and will be critical for identifying therapeutic strategies.

## 1. Introduction

Alzheimer’s disease (AD) is an age-related neurodegenerative pathology with a slowly progressive and irreversible decline of memory and of cognitive functions that involves many brain regions. AD histopathological hallmarks include the deposition of extracellular amyloid beta (Aβ) fibrils and plaques in the brain [1], and intracellular neurofibrillary tangles (NFTs).

AD is a neurodegenerative disorder characterized by atrophy of the brain, dysfunctions of neurotransmission, and loss of synapses [2,3]. AD pathophysiological mechanisms are complex and still not completely understood, although it is known that the central cholinergic neurotransmission is the first to be damaged [4], leading to progressive impairment of memory and cognition [5]. The accumulation of extracellular Aβ peptides [6,7,8], of intracellular neurofibrillary tangles (NFTs) of hyperphosphorylated tau protein [9], and neuroinflammation [10,11] are the most “classically” recognized physiopathological mechanisms of AD. Low-grade pro-inflammatory conditions that develop during aging are considered a prodrome of AD [12,13,14,15]. Franceschi and coworkers [16,17] introduced the term “inflammaging” which describes the progressive changes occurring in the aging brain, characterized by low-grade chronic up-regulation of certain pro-inflammatory responses and neuroinflammation. The association between inflammation, aging and AD is based upon complex molecular and cellular changes that are not still completely understood. A systemic increase in proinflammatory molecules induces Aβ deposition on the neuron soma [12,18], and in turn Aβ deposition triggers the NOD-like receptor family, which stimulates inflammation [13]. This mechanism of mutual reinforcement causes an increase in Aβ burden in the normal aged brain [19]. When Aβ homeostasis is lost as a result of aging or other risk factors that enhance production or aggregation of Aβ [20], or which suppress its clearance [21], oligomeric and fibrillar Aβ species begin to accumulate, and the first plaques appear. Aβ peptide first forms small soluble oligomers that grow into protofibrils and finally into dense insoluble structures that accumulate in the brain in form of extracellular Aβ plaques [22,23,24]. The hypothesis that Aβ plaques are solely responsible for the pathology of AD is widely accepted but it has not been demonstrated conclusively yet. An alternative hypothesis is that soluble Aβ species are more toxic than fibrillar Aβ and cause more profound synaptic dysfunction and neuronal loss [25,26,27,28]. Recent studies have also documented the presence of toxic oligomeric species of Aβ and tau within the AD brain, supporting the idea that these species can propagate and induce neuronal dysfunction and degeneration (for references see [29,30]). In addition to Aβ plaques, tau oligomer hyperphosphorylation causes the collapse of microtubules and forms NFTs, leading to neuronal death, cognitive decline and brain atrophy (for references see [31]). The presence of reactive glial cells within the amyloid plaques has already been described by Alois Alzheimer [32,33] and confirmed by later studies that identified both reactive astrocytes and microglia that infiltrate the Aβ deposits (among many others, [34,35,36,37]). Furthermore, there is compelling evidence that age-related vasculopaties and breakdown of the blood–brain barrier (BBB) are early causative factors in AD pathogenesis [38,39]. Indeed, lesions of the neurovascular unit are found together with the classic signs of AD such as Aβ plaques or neurofibrillary tangles in around half of dementia patients and it is becoming increasingly evident that vascular pathology is a common and early symptom in AD (see references in [40]).

In response to damaging stimuli, the brain, like any other organ, puts in place restoration mechanisms organized by numerous cells and signaling molecules. Indeed, the main function of astrocytes and microglia is protective, aimed at the preservation of the neurons, and many molecular cascades expressed by these cells are neuroprotective. Long regarded as a non-specific, mere consequence of AD pathology, activation of microglia and astrocytes is now considered a key factor, not only in protective mechanisms, but also in both the initiation and progression of the disease, and suppression of astrogliosis exacerbates neuropathology [41]. Some studies suggest that microglia dictate the profile of reactive astrocytes, but others indicate that astrocytes play a pivotal role, or that they are controlled by peripheral inflammatory cells [42,43], or that there may be a continuous multidirectional interplay between these two glia cell systems with a continuous crosstalk with neurons. This mutual interplay can form a virtuous/vicious cycle that depends on many different factors. Indeed, reactive astrocytes and microglia overexpress many cytokines, chemokines, and signaling molecules that could activate or damage neighboring cells [44,45,46]. When the equilibrium between the beneficial vs. the deleterious mode of astrocytes and microglia is altered, these cells can initiate and even exacerbate the neuropathological condition. Furthermore, there is increasing evidence of glial heterogeneity in the healthy brain [47,48,49], reinforcing the idea that different populations of glia cells may have distinct roles in disease pathogenesis. Different populations of astrocytes and microglia have mutual interactions that play critical roles not only in physiological conditions, but also in many diseases, and how cellular heterogeneity interconnects with disease is still not known. Therefore, it appears that heterogeneity of glia, either between brain regions or within a particular brain region, is likely to be relevant in healthy conditions and disease processes.

Similarly, glia–glia as well as glia–glia–neuron interactions are increasingly recognized as critical in both the healthy brain and in disease (for references see [50]). It is therefore timely to take into consideration not only how each cell type behaves in health and disease separately, but also how microglia and astrocytes talk to each other, and to neurons. This approach will give a more comprehensive account of how their dysregulation can be involved in central nervous system (CNS) injury or disease. Furthermore, many of the genes involved with increased risk of developing neurodegenerative diseases such as AD are predominantly glial genes (for references see [51]), indicating the direct involvement of glia in this pathology.

This review is focused on the description and discussion of the alterations of astrocytes and microglia, and of the changes of the interplay between neurons and glia in models of Alzheimer’s disease. In the last part, we will focus our attention on recent data that demonstrate a differential reactivity of astrocytes and microglia in CA1 and CA3 areas of the hippocampus in TgCRND8 mice, a transgenic mouse model of Aβ deposition [37].

## 2. Astrocytes Involvement in AD

Astrocytes, the most numerous glia cells in the brain, have many housekeeping functions, maintain the homeostasis of the CNS and are responsible for neuroprotection and defense (for references see [52]). Traditionally, astrocytes are divided into two major groups based on their location and structure, namely protoplasmic astrocytes and fibrous astrocytes. Protoplasmic astrocytes have a bushy phenotype, are located in the gray matter and are in direct contact with blood vessels via their endfeet. Fibrous astrocytes are located in the white matter, where they support myelination processes (for references see [53]).

In the healthy brain, astrocytes regulate the formation, maturation, and plasticity of synapses [54,55], are indispensable for neurotransmitter homeostasis [56,57], and control the formation of neural circuits [58,59,60,61,62]. Astrocytes release gliotransmitters [63,64,65,66] necessary for synaptic plasticity [65,67], and control GABA and glutamate extracellular levels at the synapses. Astrocytes mediate the synaptic functions [68,69] and are thus involved in memory formation [65,66,67,70,71]. Healthy astrocytes are fundamental cells of the neurovascular unit, and help maintaining the integrity and the functionality of the BBB and of the glymphatic system [52,68,72,73,74]. It has been proposed that vascular dysregulation and breakdown of the BBB may be the first steps in AD pathogenesis [38,39], affecting Aβ clearance [75]. Furthermore, the glymphatic system facilitates the clearance of interstitial solutes including Aβ and tau [76]. Astrogliosis causes loss of AQP4 polarization in perivascular astrocytes, which may represent a mechanism common to neurovascular unit (NVU) and glymphatic dysfunctions in many neurodegenerative diseases such as AD [77,78]. It has been shown that the glymphatic function is disrupted around microinfarcts, especially in the aging brain [76]. All these data taken together may suggest that microlesions of the neurovascular unit, also disrupting the glymphatic system, may trap proteins within the brain parenchyma, increasing the risk of amyloid plaque formation [76].

Yet, the understanding of the multiple, contrasting roles of astrocytes in pathological mechanisms entered into focus only very recently. Pathological phenotypes of astrocytes are responsible for three major responses to insults: (i) reactive astrogliosis, (ii) astroglial atrophy and loss of function and (iii) pathological remodeling [41,79].

In AD patients and in amyloid-mouse models of AD [80,81,82,83], astrocytes have high levels of GABA. In two different mouse models of AD, APP/PS1 mice (APP KM670/671NL (Swedish), PSEN1 L166P) [81] and 5xFAD mice (APPSwFlLon, PSEN1*M146L*L286V) mice [83], tonic release of GABA from hypertrophic astrocytes [81,82,83] located in the vicinity of Aβ plaques was demonstrated. At first, release of GABA from astrocytes, activating GABAA and GABAB receptors, causes a decrease in glutamate release, with a consequent decrease in excitotoxicity and neuroinflammation [84]. Later, the excess of GABA can unbalance the subtle inhibitory–excitatory equilibrium in the neuronal network, inducing inhibition of synaptic plasticity [82]. It has also been shown that astrocytes degeneration may cause the downregulation of glutamate transporters. The two most expressed isoforms of glutamate transporters in the hippocampus are EAAT-1 (Excitatory amino acid transporter-1, GLAST in rodents), and EAAT-2 (Excitatory amino acid transporter-2, GLT1) [85], mainly expressed on astrocytes. Decreased expression of either one or both glutamate transporters compromises the ability of astrocytes to reuptake the excess of glutamate, and to regulate glutamatergic transmission. This in turn results in severe excitotoxicity that underlies rapid thedevelopment of severe dementia, as shown in Wernicke encephalopathy [86,87]. In AD pathogenesis the situation seems still controversial. In AD patients, the Aβ peptide has been shown to downregulate the functional activity of glutamate transporters [88]. However, in a subsequent study, the Aβ peptide was reported to increase the cell surface expression of GLAST and augment the glutamate clearance ability of cultured astrocytes [89]. Nevertheless, it has been reported that impairment of glutamate uptake is involved in the pathogenesis of AD and other neurodegenerative disorders such as Parkinson’s disease, Huntington’s disease, and epilepsy (reviewed in detail elsewhere [90,91]).

In AD, in different brain regions and subregions, astrocytic modifications are highly heterogeneous and can result in either hypertrophy or atrophy [37,92,93,94]. In a triple transgenic mouse model of AD, Aβ plaques trigger astrogliosis, which is, however, different among brain regions. Indeed, Aβ causes hypertrophy of astrocytes mainly in the CA1 region of the hippocampus [37,95] in the entorhinal and prefrontal cortex it causes little sign of astrogliosis [96,97]. Furthermore, in the hippocampus hypertrophic astrocytes are located in close proximity to Aβ plaques, both in animal models [95] and in post mortem brain tissue from AD patients [98,99], a strategic location that is considered neuroprotective. Indeed, it has been demonstrated with PET (Positron Emission Tomography) scan in human patients that the decrease in astroglial reactivity parallels the switch from mild cognitive impairment to AD, again demonstrating the neuroprotective role of astrogliosis, at least in the prodromal phases of AD [94]. More distantly from the plaques, astrocytes look atrophic [95].

Recent studies have demonstrated that different CNS injuries stimulate at least two types of astrocytes with strikingly different properties, A1 reactive astrocytes, with detrimental properties for neurons, and A2 reactive astrocytes with beneficial, neuroprotective properties. Indeed, A2 reactive astrocytes release neurotrophic factors and cytokines that promote neuronal survival and neurogenesis, as well as synaptogenesis and repair of the damaged synapses. Among the neurotrophic factors or cytokines released by A2 astrocytes are BDNF, IL-6, CLCF1, GDF15, and thrombospondins. In addition, A2 astrocytes release gliotransmitters such as glutamate, GABA, ATP, and neuromodulators such as kynurenic and acid d-serine [100,101]. In the presence of high levels of proinflammatory cytokines, activated astrocytes increase ROS and NO production through induction of the NF-κB pathway [102]. A1 neuroinflammatory astrocytes upregulate many genes that express proinflammatory proteins and other neurodegenerative substances [100]. Recently it has been demonstrated that astrocytes in their A1 state release factors that are toxic to neurons and oligodendrocytes, and lose their phagocytic activity and possibly their ability to dispose of Aβ plaques [101]. Suppression of astroglial reactivity and phagocytosis exacerbates Aβ load and reduces neuroprotection [103].

Although astrocytes so far have been shown to acquire these two distinct reactive states, more recently it has been postulated that they may acquire many possible activated states in both the healthy and diseased brain (also see [51]). As mentioned above, these different states depend not only on the type of insult but also on the brain structure in which they are located [100]. Indeed, nine different groups of astrocytes have been defined [104]. This result possibly indicates that astrocytes acquire a reactive phenotype in function of the local microenvironment, even in healthy conditions [104].

Nevertheless, it is not understood completely yet whether astrocytes located in different cerebral structures respond to the same insult with the same morphofunctional modifications or whether they react differently to the same insult. In other words, whether astrocyte responses to injuries are controlled by intrinsic cues, or whether they depend upon external signals that come from the environment [105,106]. A third hypothesis is that there may exist a continuum in the diversity and intensity of astrocyte reaction, which possibly hides different, discrete reactive states. Recent work has demonstrated that astrocytes located in distinct anatomical regions have different molecular profiles [107,108], suggesting that astrocytes have site-specific functional roles. Astrocytes derived from different CNS regions respond differently to Aβ in vitro [109]. Indeed, this finding indicates that astrocyte heterogeneity is at least partially intrinsic, possibly due to preexisting differences between astrocytes from distinct brain regions [47,107,110,111,112,113,114]. In the mouse, hippocampus specific, age-exacerbated reactive astrogliosis causes higher vulnerability to age-related neurodegeneration [115]. For instance, an age-related morphofunctional modification of astrocytes called clasmatodendrosis, has been found in the rat hippocampus [116,117,118,119,120]. In addition, in the white matter of patients with cerebrovascular dementia and AD [121], and in patients with mixed dementia [122], astrocytes show morphofunctional modifications typical of clasmatodendrosis, which correlate directly to changes in cell function [123]. Clasmatodendrotic astrocytes show swollen and vacuolized cell bodies, shorter branches, and loss of distal processes that cause less endfeet coverage of brain vessels. These latter modifications can contribute to vascular deficits observed during aging and in AD. Furthermore, since astrocyte endfeet are main components of the BBB, their fragmentation by clasmatodendrosis can contribute to the impairment of the functionality of the barrier. Aβ clearance is essential for neuroprotection against AD, and in mouse models of AD the impairment of Aβ clearance increases neurodegeneration [124]. The deposition of high quantities of fibrillar Aβ modifies the interactions between astrocytes and neurons [117], possibly decreasing Aβ peptide disposal to the circulating system, and consequently, increasing Aβ deposition in brain parenchyma [125] that may play a significant role in neuronal damage. Therefore, clasmatodendrosis can hamper astrocyte-mediated Aβ clearance from neurons and increase fibrillar Aβ deposition [117,126].

It has been shown that Aβ reacts with receptors located on astrocytes such as CD36 (cluster of differentiation 36), RAGE (receptor for advanced glycation end products), SCARA-1 (scavenger receptor A-1), and MARCO (macrophage scavenger receptor with collagenous structure). RAGE is one of the most characterized scavenger receptors, and binding to Aβ causes proinflammatory modifications in astrocytes [127]. RAGE mediates the phagocytic profile of astrocytes [128] and the interaction with other ligands, including S100β, involved in Alzheimer disease neuroinflammation [129]. SCARA-1 is involved in Aβ clearance [108], while MARCO may decrease the inflammatory response of microglia [130], and CD36 and RAGE are implicated in the scavenging activity of microglia caused by Aβ (for references see [131]). CD36 cooperates with toll like receptors (TLR-6 and TLR-4), causing ROS production and inflammasome activation [132]. We know that expression of many proinflammatory proteins is increased in astrocytes but, interestingly, not only genes that are upregulated but also those that are down regulated may help understand the roles of reactive astrocytes in disease pathogenesis. However, no established list of down-regulated genes across multiple diseases and especially in AD so far exists [133]. To make things even more complicated, in an animal model of AD different proinflammatory proteins are expressed at different levels in astrocytes located in different areas within the hippocampus [37]. Indeed, molecular changes in astrocytes are highly context specific, with about 50% of modified gene expression that depends on the type of brain damage [134].

It has been shown that astrocytes can participate with microglia in phagocytic events [135,136,137,138,139]. Astrocytes use the ABCA1 [138], MEGF10, and MERTK [140], as well as BAI1 and integrin αvβ3 or αvβ5 [141] pathways for phagocytosis. Since astrocytes are not as mobile as microglia [142,143], they are not able to migrate, but polarize their distal processes, and engulf apoptotic bodies derived from dendrites of dying neurons or other toxic material such as Aβ. Astrocytes and microglia play orchestrated roles in a highly coordinated way, with differences in different brain areas that can have important physiopathological consequences [138]. Reactive astrocytes have dual roles in Aβ plaque degradation. The phagocytic role of reactive astrocytes in amyloid pathology may contribute to the clearance of dysfunctional synapses or synaptic debris, thereby restoring impaired neural circuits and reducing the inflammatory impact of damaged neurons [144].

Notwithstanding all this new evidence, the role of astrocytes in AD is still controversial. On one side, astrocytes are able to remove Aβ fibrils from neuron membranes [126] for their disposal [125]. On the contrary, it has been demonstrated that astrocytes may also contribute directly to Aβ peptide overproduction especially in the presence of increased cellular stress caused by environmental factors and increased neuroinflammation [126,145,146,147,148,149]. On the other side, a decrease in the size of astrocytes and reduction in the number of GFAP-positive primary branches is observed in the hippocampus, prefrontal cortex, and entorhinal cortex at the early stages of the pathology in mouse models of AD [95,96,97,150]. These phenotypic modifications can possibly cause decreased Aβ disposal and increased Aβ extracellular levels.

In the healthy brain, astrocytes are organized in non-overlapping domains while reactive astrocytes lose their domain organization. The significance of astrocytic domains in health and their spatial dysregulation in disease remains unclear. Many chronic neurological disorders are accompanied by chronically stressed, degenerated, and atrophic astrocytes with loss of function, which adds to the progression of the disease. At the early stages of AD, gliosis is markedly increased, and reactive astrocytes are located around Aβ plaques [95,151,152], while large numbers of astrocytes undergo atrophy [153]. In these conditions, astrocytes undertake a series of phenotypic and functional changes [154] that lead to the formation of a sort of scar around the plaque. Scar formation starts as a defensive reaction aimed at the isolation of the plaque from the healthy tissue for neuron survival. To this aim, astrocytes release neuroprotective agents such as BDNF, VEGF, CLCF1, thrombospondins and bFGF, or IL6 and GDF15 [154].

## 3. Microglia Involvement in AD

Microglia, the primary immune cells of the central nervous system, patrol the brain parenchyma as sensors, to detect and eliminate debris or apoptotic neurons by phagocytosis [155], and to restore tissue homeostasis. It has been shown [142,156] that microglia have very mobile ramified branches that allow a dynamic and continual survey of the parenchyma and phagocytosis of damaged neurons. Microglia actively maintain their protective role during normal aging [157,158,159,160] by clearing dying neurons [161], but this ability is considerably decreased in a proinflammatory context [155]. In parallel, in an animal model of AD the phagocytic activity and clearance capacity of microglia inversely correlate with Aβ plaque deposition and aging [162].

It is known that microglia exist in different phenotypes (for references see [163]). The proinflammatory M1 state occurs when microglia, activated after an acute insult, release proinflammatory cytokines such as TNFa, IL-1, IL-6, IL-18. The M2 non-inflammatory state of microglia is associated with secretion of anti-inflammatory cytokines such IL-4, IL-10, IL-13 and TNF-ß. Nevertheless, this quite recent classification of microglia in M1 and M2 states [100] seems to be rather narrow, not corresponding to the variety of microglia phenotypes so far discovered in the brain [164,165]. It is becoming evident that each subtype of microglia has intrinsic heterogeneity, displays intrinsic properties and performs unique functions [164,165]. Furthermore, the microglia activation profile is not an all-or-none phenomenon but rather a continuum of different levels of activation states, which depend on the type of insult and its progression, as well as on the area(s) of the brain where microglia cells are located [48,166]. Data from animal models of AD show that activated microglia are recruited at the site of Aβ deposition, interact with Aβ deposits and regulate Aβ levels in the brain [167,168].

The role of microglia in neurodegenerative disorders such as AD is influenced by the expression of apolipoprotein E (APOE) and triggering receptor expressed on myeloid cells 2 (TREM2) [169]. In acute models of neurodegeneration, it has been demonstrated that APOE regulates TREM2, which in turn modulates the activation of microglia [169]. TREM2, by sustaining cellular energetic and biosynthetic metabolism, has been also linked to the regulation of microglia metabolism [170], which has a key role in maintaining the high microglial activity needed to deal with the excess of amyloid deposition. This effect has been linked to the more intense microglia response to plaque formation in AD, which in turn causes a harmful chronic inflammatory response [171]. Variants of TREM2 impair phagocytic properties, inflammatory responses, energy metabolism, plaque compaction and activation of microglia, affecting the progression of AD [170,172,173]. In addition, toll like receptor 4 (TLR4), expressed on microglia, plays an important role in neuroinflammation [174]. Nevertheless, studies on TLR4 and on TREM2-deficient mice give conflicting results on AD pathology [175]. TREM2-deficient APP-PS1 mice display reduced accumulation of microglia around plaques and TLR4, which can be stimulated by both fibrillar and oligomeric forms of Aβ [176], has also been suggested to be protective in AD [177,178]. Further, activation of toll like receptor 2 (TLR2), which can be stimulated by fibrillar Aβ, activates microglia into a more pro-inflammatory profile, with detrimental effects on AD pathology [176]. Therefore, activated microglia perform phagocytosis of Aβ deposits, contributing to Aβ clearance and removal of cytotoxic debris from the brain [157,179,180,181,182,183]. Indeed, plaque-associated microglia cells exhibit signs of uptake of the Aβ peptide, giving evidence that they can inhibit additional fibrillization of Aβ and plaque growth [184], thus protecting neighboring neurons [185]. Furthermore, in TREM2 deficient mice, loss of microglia clustering around Aβ plaques increases AD risk, supporting the idea that microglia can have a protective effect, decreasing AD pathogenesis [185], as also shown in PS2APP (PS2N141I x APPswe) mice in which continuing microglial response seemed to impart preserved cognitive performance [186]. Nevertheless, sustained activation of microglia can increase Aβ deposition, phagocytosing healthy neurons [181,187,188,189,190] and intensifying neurodegeneration [12,17]. The phagocytosis of whole neurons that show no sign of neurodegeneration [174] is defined as phagoptosis (also called primary phagocytosis). Phagoptosis was first described by Brown and Neher [191] and defined as ”death caused by being devoured”. It is triggered by a stimulus which is too mild to cause neuronal death per se but too intense to allow the neuron to recover, and sufficient to release “find-me” signals that activate and recruit microglia and astrocytes for phagocytosis [191,192,193]. Microglia associated with Aβ plaques show a neurodegenerative phenotype, regulated by the TREM2-APOE pathway, which suppresses the phagocytosis of apoptotic neurons [169]. Microglia projections are chemotactic sensors that extend towards injured cells in the “find-me” step of neuron phagocytosis [157]. Their age-related impairment may weaken the neuroprotective activity of microglia. Reasonably, decreased microglia migration may hamper its phagocytic efficacy, thus favoring the accumulation of degenerating neurons and proinflammatory toxic debris [194], typical of brain aging [116]. Indeed, microglia phagocytoses Aβ fibrils less efficiently in aged mice than in young mice [195]. Nevertheless, it is still accepted that amplified, exaggerated, or chronic microglia activation can lead to robust pathological changes and neurobehavioral complications such as in chronic inflammatory diseases [196] (for references see also [197]). Furthermore, it has been demonstrated in animal models of AD that microglia, in response to soluble Aβ, can phagocytose synapses [198], while depletion of microglia prevents loss of neurons and dendritic spines [199], further suggesting a pathogenic role for microglia. Furthermore, senescence of microglia is thought to contribute to neurodegenerative disorders [200]. Proliferation of microglia in a mouse model of β-amyloidosis increases three times in comparison to controls and microglia priming early in life can induce functional changes that may contribute to age-related neurodegenerative diseases [201].

Nevertheless, examples exist of positive effects of active microglia and production of cytokines in early brain development [202], in the support, synaptic pruning [203] and in normal memory and learning [204]. A unique, novel subtype of protective microglia, disease-associated microglia (DAM), has recently been described [166]. DAM cells have enhanced phagocytic activity in both AD transgenic mice and human AD brains [166]. DAM cells express genes that encode a large number of factors that contribute to disease mitigation [166]. As reported by Keren-Shaul and colleagues [166], the function of the genes expressed by DAM are first TREM2-independent and later TREM2-dependent. The transition to fully-activated DAM does not occur in the absence of the TREM2 receptors, supporting the idea that TREM2 is necessary to mitigate the disease by supporting phagocytosis at late stage of the disease. The function of TREM2 during the first period of AD is the phagocytosis of Aβ peptides, with no evident inflammation [166,205]. An increased level of TREM2 during this stage is a defensive factor linked to Aβ clearance [166,206]. In more advanced stages of the AD, the role of TREM2 evolves. TREM2-expressing microglia cause extensive inflammation and neurodegeneration interacting with accumulating NFT [207,208]. Corroborating this, is the observation that the absence of TREM2 in microglia at the late stage of AD, but not at the early stage, exacerbates the disease symptomatology [209,210]. Complement C3 is one of the most highly upregulated genes involved in this reaction [108] from microglia, also responsible for excessive release of proinflammatory mediators and induction of proliferation of A1 reactive astrocytes [101]. It appears, therefore, that none of the genes expressed by DAM cells represents the primary cause of the disease but, rather, they affect AD time-course and progression rate. Distinct microglia subpopulations may have different roles at different times in disease progression [166], and may be located in different brain areas, but further work will be needed to dissect out these features. Microglial activation does not develop in an all-or-none fashion; rather it develops as a continuum, time- and space-dependent phenotypic remodeling, typically associated with neuroprotection and maintainance of brain homeostasis. The modifications of microglia states are generally fully and rapidly reversible [156].

Boosting microglia defensive capabilities with cell-specific therapies may offer new avenues for preventing or reversing neurodegeneration.

## 4. Astrocytes-Microglia Crosstalk in AD Mechanisms

Glia cells and neurons communicate with each other in both health and disease conditions (for further references see [211]). Astrocytes–microglia crosstalk is maintained via growth factors, gliotransmitters, cytokines, chemokines, innate-immunity mediators, ATP, mitogenic factors, NO, ROS, and glutamate. ATP derived from astrocytes, binding to purinergic P2Y12 and P2Y6 receptors expressed on microglia, promotes microglial processes extension and phagocytosis [212]. Microglia express and release cytokines, such as IL-1β, TNF-α, and IL-6, which regulate astrocytic responses and decrease P2Y1 receptors on astrocytes to enable tissue remodeling and repair [213]. Astrocyte–microglia interplay through activation of the complement is fundamental to the modulation of Aβ pathology and neuroinflammation in mouse models of AD [108]. Aβ induces expression of complement factor C3 from astrocytes [214]. Astrocyte-secreted C3 interacts with the C3a receptor (C3aR) on microglia to regulate microglial phagocytosis [214]. Furthermore, cleavage products of C3, such as C3a, C3b, and iC3b mediate phagocytosis of Aβ by microglia [215,216,217]. A different set of cytokines produced by activated microglia, such as IL-1α, TNF-α and complement factor C1q, induce in astrocytes a neurotoxic state.

Furthermore, astrocytes, microglia, and neurons intercommunicate via extracellular vesicles called exosomes [218] that can contain mRNA [219] and miRNA [220] (for further references see [221]), capable of modulating gene expression in distant cells.

Recent work from Barres’s lab demonstrates that activated microglia cells adapt their secretory profile increasing the release of factors such as C1q, TNF-α, IL-1α, influencing astrocyte activation during inflammatory responses [101]. When microglia are close to astrocytes, they establish many contacts with astrocytes branches, where the mechanosensor integrin-b1 is highly accumulated [222]. Indeed, integrin-b1, specifically localized in high density at the contact sites microglia/astrocytes, possibly controls the dynamic remodeling of microglia branch tree [222], as has also been demonstrated in the spinal cord [223]. In CA1 of aged rats, it is demonstrated that microglia branches are smaller and shorter and have lower expression of IBA1 than those of young rats [222]. As a consequence, it appears that microglia have impaired mobility and phagocytic activity, which results in the accumulation of neuronal debris [116,224] and toxic compounds that can start a vicious cycle of neurodegeneration. Microglia cells in the proximity of disrupted astrocyte branches, such as those of clasmatodendrotic astrocytes, show amoeboid morphology, have shorter and enlarged projections, and have lower accumulation of integrin-b1 [222]. These data suggest that impairment of the direct interaction between astrocytes and microglia may hamper branching and migration of microglia, with consequent lower mobility, and less scavenging capacity of these cells.

It has been demonstrated in two different APP transgenic mouse models that almost complete ablation of resident microglia does not alter Aβ plaque load [225] extending and confirming previous work [226], and suggesting that resident microglia are not important in the de novo formation of amyloid [225].

## 5. Region-Specific Modifications in AD

Many neurological conditions, including AD, occur in a region-specific way and have differential regulatory mechanisms of gene expression [227]. Alzheimer’s disease primarily affects the hippocampus and cortex, damaging and destroying the connections between neurons and later causing cell death. Although initial symptoms are minor, this damage leads to impairments in learning, memory, and thinking, and is eventually fatal [228,229,230].

In both animal models and in AD patients, CA1 is the most vulnerable region of the hippocampus to neuronal loss [231,232,233,234,235,236,237], while CA3 is a lesser damaged area [238,239]. Among AD patients, a variable degree of atrophy between CA1 and CA3 is often found, CA3 being the least damaged area [238,239]. Nevertheless, no conclusive explanation on the differential sensitivity of CA1 has so far been given. Therefore, to study and compare the results obtained in these two hippocampal areas is fundamental, and can help explaining the more pronounced sensitivity of CA1 pyramidal neurons to neurodegenerative insults, both in experimental animal models and in humans [236,237,240,241,242].

The hippocampus is often described as a unitary structure formed by distinct areas, but it is becoming evident that this is hardly the case. The unique molecular and synaptic milieu of its spatial domains in CA1 in comparison to CA3 lead us to ask how AD pathophysiology can be more prominent in one region versus the other one. The hippocampal areas CA3 and CA1 have distinct functions, contributing uniquely to specific information processing such as novelty detection, encoding, short-term memory, intermediate-term memory and retrieval [243]. CA3 is involved in processes associated to rapid formation of spatial or contextual memory [244,245,246,247], whereas CA1 is critical for mediating associations with temporal components, serving as a “holding memory”, capable of maintaining short-term memory representations [248].

It has been proposed that functional and morphological changes of neurons in the hippocampus are associated with changes in microglial cell response during AD progression [249]. Significant progress has been made in understanding the relationships of amyloid pathology with hippocampal dysfunctions, but complete understanding of this process across hippocampal anatomical areas remains incomplete. Memory impairment, particularly episodic and spatial memory, is the most important symptom of AD, often related to the dysfunction of pyramidal neurons in CA1 and the entorhinal cortex [231,238,250,251]. Our recent data [37] lend support to the idea that the Aβ load exerts greater effects on CA1 than on CA3. However, in different transgenic mouse models of AD, it has also been found that learning and memory deficits are not directly correlated to Aβ load [252]. Therefore, other factors, besides Aβ deposition, may be involved in CA1 pyramidal degeneration and memory deficits.

## 6. Differential Patterns of Glia Activation and Neurodegeneration in CA1 and CA3 Hippocampus

In recent years, it has become more and more evident that neurodegenerative processes manifest with differential, regional-specific patterns in the brain. This heterogeneity is the expression of the diverse sensitivity and response of neurons and glia to a noxious stimulus in the different regions and subregions of the brain. Moving in this framework, and to investigate the differential susceptibility of different brain areas to insults, we investigated animal models of neurodegenerative conditions such as normal brain aging, LPS-induced neuroinflammation, brain chronic hypoperfusion, brain focal ischemia and AD [37,116,117,222,224,253,254,255,256].

**Figure 1:**
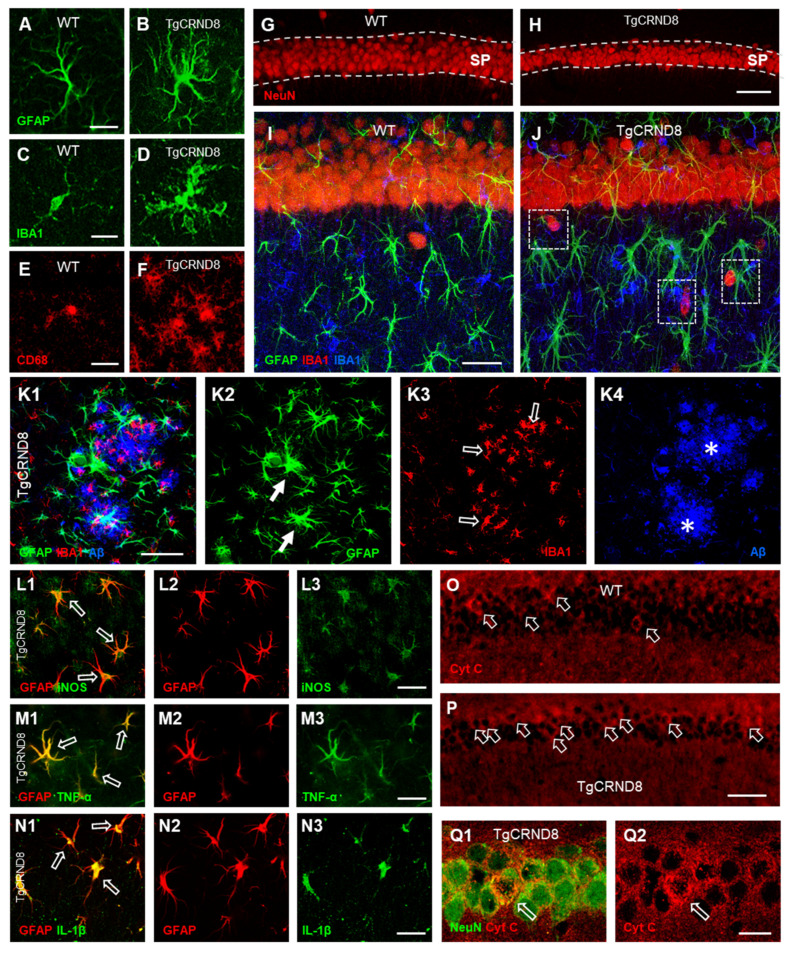
(**A**,**B**) Representative confocal photomicrographs of GFAP immunostaining of an astrocyte (green) in CA1 SR of a WT (**A**) and a TgCRND8 mouse (**B**). Scale bar: 15 µm. **(C**,**D)**: representative confocal photomicrographs of IBA1 immunostaining of microglia (green) in CA1 of a WT (**C**) and a TgCRND8 mouse (**D**). Scale bar: 20 µm. (**E**,**F**): Representative confocal photomicrographs of CD68 immunostaining of reactive microglia (red) in CA1 SR of a WT (**E**) and a TgCRND8 mouse (**F**). Scale bar: 20 µm. (**G**,**H**) Representative confocal photomicrographs of NeuN immunostaining of neurons (red) in CA1 SP of a WT (**G**) and a TgCRND8 mouse (**H**). Scale bar: 60 µm. (**I**,**J**) Representative confocal photomicrographs of triple immunostaining of neurons (NeuN, red), astrocytes (GFAP, green), and microglia (IBA1, blue) in the CA1 SR of a WT (**I**) and of TgCRND8 mouse (**J**). Neuron–astrocyte–microglia triads are evidenced in (**J**) (framed areas). Scale bar: 40 µm. (**K1**–**K4**): representative confocal photomicrographs of triple immunostaining of astrocytes ((**K2**), green, white arrows), microglia ((**K3**), red, open arrows), and amyloid beta (Aβ) plaques ((**K4**), blue, asterisks) in CA1 SR of a 6 months old TgCRND8 mouse. The merge is shown in panel (**K1**). Scale bar: 50 µm. (**L1**–**L3**) Double staining immunohistochemistry with anti iNOS ((**L3**), green) and anti GFAP ((**L2**), red) antibodies in CA1 SR of a TgCRND8 mouse. iNOS is colocalized in astrocytes ((**L1**), open arrows). Scale bar: 25 µm. (**M1**–**M3**) Double staining immunohistochemistry with anti TNF-α ((**M3**), green) and anti GFAP ((**M2**), red) antibodies in CA1 SR of a TgCRND8 mouse. TNF-α was colocalized in astrocytes ((**M1**), open arrows). Scale bar: 25 µm. (**N1**–**N3**) Double staining immunohistochemistry with anti IL-1β ((**N3**), green) and anti GFAP ((**N2**), red) antibodies in CA1 SR of a TgCRND8 mouse. IL-1β is colocalized in astrocytes ((**N1**), open arrows). Scale bar: 25 µm. (**O**,**P**): representative photomicrographs of Cyt C immunostaining of apoptotic neurons ((**O**,**P**) red) in CA1 SP of WT and a TgCRND8 mouse. The open arrows point to apoptotic neurons in CA1 SP. Scale bar: 100 µm. (**Q1**,**Q2**) Confocal magnification of Cyt C positive apoptotic neurons ((**Q2**), red, open arrow) in CA1 SP of a TgCRND8 mouse. Scale bar: 30 µm. (Modified from [37]).

Investigating the transgenic mouse model of Aβ deposition [37], we postulated that differential patterns of neurodegeneration are at the basis of the more pronounced sensitivity of CA1, found in AD and in other pathological conditions in animal models and in humans [240,241,242]. The comparison of plaque deposition, glia activation, inflammatory markers in CA1 and CA3 can help to explain their different functional, structural, and morphological alterations in AD [257] and the higher sensitivity of CA1 pyramidal neurons to insults, found in both experimental animals and humans [240,241,242]. Using an animal model of Aβ deposition, the double transgenic TgCRND8 mouse, expressing a double mutant form of human APP (K670/M671L and V717F) [36,258], and dissecting the hippocampus in areas CA1 and CA3 and into their subregions, it was possible to study plaque deposition, glia activation, inflammatory markers and neurodegeneration. The quantitative and morpho-functional alterations of neurons, astrocytes, microglia, as well as of markers of apoptosis and inflammation were evaluated taking advantage from the method of triple labelling fluorescent immunohistochemistry coupled to confocal microscopy (TIC). Most of the parameters involved in the pathophysiological mechanisms of AD, such as plaque deposition, astrocytes and microglia activation, as well as neuron degeneration show area-dependent differences [37]. Figure 1 summarizes the data obtained, that depict a heterogeneous reactive milieu in the two hippocampal subregions.

The pattern of distribution of the Aβ plaques [37] show that medium and large plaques are significantly more numerous than small plaques in CA1 than in CA3. This result indicates that Aβ fibrils in CA1 are either more actively secreted or less efficiently disposed of and continue to accumulate in the parenchyma, enlarging the amyloid plaques that over time become complex. Aβ plaques induce the production and release of pro-inflammatory cytokines by neurons and astrocytes [183,259], which promote a shift in microglia activity, from surveillance/maintenance mode, to execution of immune tasks. Although studies on appearance and development of Aβ plaques have yielded inconsistent results [184,226,260], according to Serrano-Pozo et al. [261], the average plaque size varies among individuals and correlates with age of symptoms onset, allowing speculation that large plaque size predisposes an individual to early AD onset. It has also been shown that the radius of Aβ plaques grows constantly over 6 months in mice, showing that the amount of soluble Aβ is the primary and limiting factor in plaque growth [262], and indicating a critical relationship between Aβ concentration in the parenchyma and Aβ plaque growth [260]. Aβ is added continuously in layers to the existing plaques during the presence of excessive soluble Aβ [260,262]. Additional evidence shows hippocampal subregional-specific patterns of neurodegeneration at different stages of Aβ deposition [263,264]. Nevertheless, the question arises as to why contiguous regions of the hippocampus have such significant differences in terms of plaque load. One different hypothesis may lie in the results recently published by Pascoal and collaborators [265]. They demonstrated in subjects with mild cognitive impairment and in transgenic rats that distant Aβ induces regional metabolic vulnerability, whereas the interaction between local Aβ with a vulnerable environment drives the clinical progression of dementia [265].

Hypertrophic astrocytes as well as activated microglia are located preferentially around large Aβ plaques (Figure 1(K1–K4)), in agreement with data obtained by Bolmont et al. [184] in the cortex of APPPS1 transgenic mice. Astrocytes and microglia located more distantly from the plaques are in a less reactive state [37]. Nevertheless, the specific reactive phenotype of microglia [266] and astrocytes [95] is determined not only by the proximity to plaques, but also by the size of plaque and by the distribution within different brain areas. The involvement of astrocytes in AD progression depends upon the brain area involved and the severity of the disease. Indeed, in the hippocampus, AD progression has been associated with early atrophy of astrocytes [267,268] that, at later stages of the disease, coexists with reactive astrocytes around plaques [95,97]. Nevertheless, differently from CA3, high levels of astrogliosis are present in CA1, evidenced by increased recruitment of strongly reactive astrocytes, with long and hypertrophic branches (Figure 1A,B). Furthermore, the proinflammatory mediators TNF-α, IL-1β, as well as iNOS increase in CA1 astrocytes, and only to a lesser extent in CA3 [37]. In models of AD, TNF-α usually is expressed in the brain mainly by activated microglia, and less by activated astrocytes and neurons [254,269,270]. Aβ deposition activates astrocytes and induces increased expression and release of cytokines, interleukins, NO and other proinflammatory mediators [11,271]. Increased expression and release of TNF-α by astrocytes activates cell-surface TNF-α type I receptors containing death domains [183,259,272], and increases pro-apoptotic cascades in a significant number of pyramidal neurons [270], exacerbating AD pathology [273]. In addition, cytokines can stimulate iNOS in astrocytes [37], increasing the release of NO, which is toxic to neurons. It has been demonstrated that iNOS is upregulated in the brain of AD patients [274], and iNOS KO in mouse models of AD results in a protective effect towards the pathology [275]. Many studies have shown that the gradual deposition of Aβ peptides and overproduction of inflammatory mediators activate astrocytes, further inducing the expression and release of cytokines, interleukins, NO and other proinflammatory mediators [11,271].

In the hippocampus of transgenic mice and in AD patients, reactive astrocytes surrounding Aβ plaques enwrap and engulf axonal synapses [144] and endocytose extracellular monomeric and oligomeric Aβ [276]. When astrocytes engulf large amounts of Aβ protofibrils, incomplete digestion results in intracellular load of high levels of partially truncated toxic Aβ, which can cause severe lysosomal dysfunction [277]. Accumulation of Aβ by astrocytes gives rise to enlarged extracellular microvesicles that contain N-terminally truncated Aβ and can induce apoptosis of neurons [277].

Total microglia and reactive M1-like microglia increase in both CA1 and CA3 of transgenic mice (Figure 1C–F) and are polarized towards plaques [37]. It appears that the inflammatory milieu triggered by plaque deposition recruits microglia and increases its reactivity. Microglia activation may exacerbate inflammation, increase Aβ-deposition, and intensify neurodegeneration [278]. As pointed out above, microglia can assume two different phenotypic forms, M1 and M2 [279]. While M1 microglia express and release proinflammatory cytokines [280,281], M2 microglia, phagocytosing apoptotic or degenerating neurons, is active in the surveillance of the parenchyma and maintenance of tissue homeostasis, preventing secondary inflammatory mechanisms and promoting tissue regeneration [282,283]. In AD, as for astrocytes, pro-inflammatory and detrimental, or anti-inflammatory and even protective properties have been attributed to microglia [11,200,284].

All these different findings may suggest that microglia may acquire heterogeneous activation states, and microglia can be protective or detrimental, depending on the region where they are located. Increased reactivity state of microglia and astrogliosis augment the formation of neuron–astrocytes–microglia triads in CA1. The concerted actions of astrocytes and microglia in the formation of triads with neurons help recognize danger signals, including cellular debris produced from apoptotic or necrotic cells [285], and help to dispose of damaged neurons or neuronal debris by phagocytosis [116,253]. Degenerating neurons are engulfed by microglia, and reactive astrocytes cooperate in the phagocytic event, infiltrating and dissecting the body of the damaged neuron and forming a microscar around it, possibly to prevent the spread of noxious neuronal debris in the tissue (Figure 1J).

In addition, neurons respond differently in CA1 and CA3 to the insult caused by Aβ deposition: in CA1, pyramidal neurons are significantly smaller than in controls (Figure 1G–J), many of them are apoptotic (Figure 1(O–Q2)), and their number is decreased, while in CA3 these alterations are significantly less frequent. The loss of CA1 pyramidal neurons, which undergo neuronal death by apoptosis, causes shrinkage of the CA1 pyramidal cell layer. All these modifications may be at the basis of memory loss which has been repeatedly demonstrated in this transgenic mouse model of Aβ deposition, even at early stages of Aβ deposition [258] or in the absence of plaques [286], at a stage in which microglia are activated. Indeed, microglia activation during the early phases of Aβ deposition may be deleterious due to potential adverse effects associated with inflammation, neurotoxicity and degeneration. It has been demonstrated that activated microglia can release proinflammatory cytokines such as IL-1β, IL-6 and TNFα, and generate ROS that enhance oxidative stress [287,288]. Furthermore, activated microglia can stimulate synaptic pruning and the phagocytic activity of live neurons [188], thus increasing neurodegeneration and impairing synaptic function [181]. Furthermore, neuroinflammation can enhance Aβ accumulation through perturbations of microglia phagocytic clearance of Aβ, thus enhancing Aβ deposition [289]. Furthermore, recent studies have demonstrated that microglia may play an important role in regulating astrocytes activation. Indeed, fragmented mitochondria released from activated microglia trigger the A1 astrocytic response and propagate inflammatory neurodegeneration [290]. A1 astrocytes have a reduced ability to support neuronal survival, outgrowth and synaptogenesis and can induce cell death in neurons [101,290].

All these mechanisms taken together may be the cause of the significant loss of CA1 pyramidal neurons and shrinkage of CA1 stratum pyramidale [37]. The loss of CA1 pyramidal neurons is caused—at least in part—by increased apoptosis, more pronounced in CA1 than in CA3. In CA1, the proinflammatory milieu above described intensifies pro-apoptotic cascades possibly targeted by increased TNF-α, expressed and released by astrocytes (Figure 1(M1–M3)) [270,272], and exacerbating AD pathology [273]. In addition, stimulation of iNOS by cytokines in astrocytes (Figure 1(L1–L3)) may cause increased release of NO that can be toxic to neurons. Indeed, iNOS is upregulated in AD patients’ brains [274] and iNOS KO is protective in mouse models of AD [275].

Characterizing the quantitative and morpho-functional alterations of neurons and glia in the CA1 and CA3 subregions of the hippocampus, it was possible to confirm the existence of differential and regional-specific patterns of glia activation that possibly mirror and/or cause neurodegeneration. All these mechanisms, taken in the perspective of other published results [116,117,222,224,253,254,255,256] can help explaining the higher sensitivity of CA1 pyramidal neurons in AD.

## 7. Conclusions

**Figure 2:**
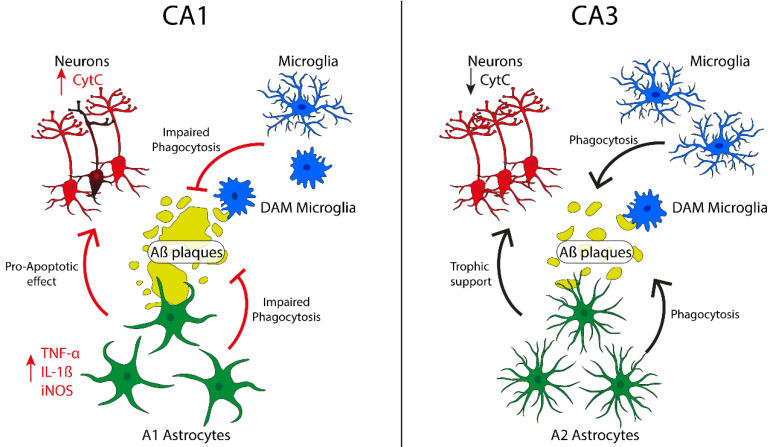
Schematic representation of the regional differences in CA1 and CA3. DAM: disease-associated microglia.

These findings allow us to shed light on the different sensitivity and response of communicant and tightly interconnected regions toward noxious stimuli or conditions such as Aβ plaque formation (See Figure 2).

Astrocytes in their A1 state release factors that are toxic to neurons and oligodendrocytes, become less synaptogenic and lose their phagocytic activity and possibly their ability to dispose of Aβ plaques [101]. This latter mechanism can be the cause of the prevalence of medium and large plaques in CA1. High levels of Aβ that form medium and large plaques can stimulate the release of pro-inflammatory cytokines by neurons and astrocytes [183,259] which promote the shift of microglia from surveillance/maintenance mode (M2), to execution of immune tasks (M1). In both CA1 and CA3 of transgenic mice microglia cells are polarized towards large Aβ plaques [37], indicating that the inflammatory milieu triggered by plaque deposition can cause increased recruitment of microglia, and significant increase in its reactivity. It is now thought that microglia undergo phenotypic activation in response to fibrillar beta-amyloid (fAβ) deposits that form amyloid plaques. However, despite the presence of abundant plaque-associated microglia in animal models of the disease [37] and in the brains of AD patients, it has been reported that microglia fail to efficiently clear fAβ deposits [155,166]. Recent transcriptomics studies have shown that in neurodegenerative diseases, chronic neuroinflammatory states and in advanced aging microglia gradually adopt a unique phagocytic DAM phenotype [166,291,292,293]. DAM microglia first maintain Aβ plaques in a benign state, expressing factors that prevent tau hyperphosphorylation and neuronal damage [166]. Nevertheless, the existence of both pro-inflammatory and anti-inflammatory DAM microglia has been demonstrated, with consequent potential benefits of suppressing the pro-inflammatory DAM phenotype [294]. Indeed, in more advanced stages of AD, TREM2-expressing microglia cause extensive inflammation and neurodegeneration [207,208]. Microglia can assist the clearance of age related Aβ accumulation or can promote inflammatory reaction to it, eventually causing widespread neurodegeneration. This difference can be driven not only by the time-course of the disease, but also by spatial cues.

Microglia can be seen also neuroprotective or neurotoxic depending also on the crosstalk with astrocytes. Indeed, the concomitant presence in CA1 of A1-type reactive astrocytes, which lose their ability to phagocytose damaged neurons [138] can start a vicious cycle in which microglia that has lost its scavenging activity is no longer able to dispose of the amyloid fibrils that add up making plaques bigger and bigger and less disposable. This is in line with recent evidence that demonstrates subregional-specific patterns of neurodegeneration at different stages of Aβ deposition [263,264].

In CA1 and CA3, contiguous and interconnected regions of hippocampus, astrocytes and microglia show differential, finely regulated, and region-specific reactivities, which are different depending upon the physiopathological conditions. Whether glial cells adopt phenotypes that aggravate tissue injury or promote brain repair, most likely depends on different set of factors, such as the nature of the damaging element, the time course of injury, the severity score that determine the precise arrangements of signals deriving from the surrounding environment. Therefore, the response is possibly not univocal but largely depends on the disease context.

The idea that the same stimulus/injury activates different types of astrocytes/microglia cells in different regions of the brain raises many questions. How many reactive astrocytes/microglia cell types are there? What are the cell–cell interactions that induce reactive astrocytes and/or microglia? What are the relevant extracellular and intracellular signaling pathways that induce reactive astrocytes/microglia? In the same way that microglia can have simultaneously multiple profiles of activation, the A1 and A2 phenotypes can represent the extremes of a continuous spectrum of reactive profiles. The mechanisms regulating these diverse functional properties remain unknown, but evidence suggests that environmental cues, especially microglia-derived signals [101,295] may be important.

A complete understanding of the spatial differences and roles of glia will allow us to assess how these interactions can influence the disease state, the progression of disease, and will be critical to identify therapeutic strategies for recovery.

## Abbreviations

Aβ	Beta amyloid
ABCA1	ATP-binding cassette transporter
AD	Alzheimer’s disease
APOE	Apolipoprotein E
APP/PS1 mice	APP KM670/671NL (Swedish), PSEN1 L166P
BAI1	Brain-specific angiogenesis inhibitor 1
bFGF,	Basic fibroblast growth factor
BDNF	Brain-derived neurotrophic factor
CA1	Cornu Ammonis area 1
CA3	Cornu Ammonis area 3
CD36	Cluster of differentiation 36
CLCF1	Cardiotrophin-like cytokine factor 1
CNS	Central nervous system
CytC	Cytochrome C
DAM	Disease activated microglia
5xFAD mice	APPSwFlLon, PSEN1*M146L*L286V
GABA	Gamma aminobutyric acid
GDF15	Growth/differentiation factor 15
GFAP	Glial fibrillary acidic protein
GLAST	Excitatory amino acid transporter-1, EAAT-1
GLT1	Excitatory amino acid transporter-2, EAAT-2
IBA1	Ionized calcium binding adaptor molecule 1
IL-1	Interleukin 1
IL-1ß	Interleukin 1 ß
IL-6	Interleukin 6
IL-10	Interleukin 10
IL-13	Interleukin 13
IL-18	Interleukin 18
iNOS	Inducible Nitric Oxide synthase
LPS	Lipopolysaccharide
MEGF10	Multiple EGF like- domains 10
MERTK	Proto-oncogene tyrosine-protein kinase MER
NeuN	Neuronal nuclei
NFT	Neurofibrillary tangle
NO	Nitric Oxide
NF-κB	Nuclear factor kappa-light-chain-enhancer of activated B cells
NVU	Neurovascular unit
PET	Positron Emission Tomography
PS2APP mice	PS2N141I x APPswe
ROS	Reactive oxygen species
TIC	Triple-labeling fluorescent immunohistochemistry coupled with confocal microscopy
TLR4	Toll like receptor 4
TNF-α	Tumor necrosis factor-α
TNF-ß	Tumor necrosis factor-β
TREM2	Triggering receptor expressed on myeloid cells 2
VEGF	Vascular endothelial growth factor
